# Neutralizing Autoantibodies to Type I Interferons in COVID-19 Convalescent Donor Plasma

**DOI:** 10.1007/s10875-021-01060-0

**Published:** 2021-05-19

**Authors:** Sara E. Vazquez, Paul Bastard, Kathleen Kelly, Adrian Gervais, Philip J. Norris, Larry J. Dumont, Jean-Laurent Casanova, Mark S. Anderson, Joseph L. DeRisi

**Affiliations:** 1 University of California, San Francisco, CA USA; 2 Laboratory of Human Genetics of Infectious Diseases, Necker Branch, INSERM U1163, Necker Hospital for Sick Children, Paris, France; 3 University of Paris, Imagine Institute, Paris, France; 4 St. Giles Laboratory of Human Genetics of Infectious Diseases, Rockefeller Branch, The Rockefeller University, New York, NY USA; 5 Vitalant Research Institute, Denver, CO USA; 6 University of Colorado School of Medicine, Aurora, CO USA; 7 Geisel School of Medicine at Dartmouth, Lebanon, NH USA; 8 Howard Hughes Medical Institute, New York, NY USA; 9 Chan Zuckerberg Biohub, San Francisco, CA USA

To the Editor:

Convalescent plasma has been a mainstay therapeutic in passive immunization for decades. In the setting of the novel coronavirus (SARS-CoV-2), it has become widely used for prophylaxis and early intervention in COVID-19 [1*, *2]. Recently, neutralizing autoantibodies to type I interferons have been described in at least 10% of patients with critical COVID-19 pneumonia, while they were absent from infected individuals with asymptomatic or mild disease [3]. These autoantibodies are likely pre-existing and have an immunological impact early in the course of COVID-19 [3,4].

Given the role of auto-Abs to type I interferons in the development of life-threatening COVID-19 pneumonia, we determined the prevalence of anti-type I interferon antibodies in the convalescent plasma supply from a large blood bank. We selected 116 convalescent plasma samples from unique donors who had previously been hospitalized for COVID-19, and found that 4/116 (3%) plasma samples were positive for anti-IFN-α2 autoantibodies (Fig. 1a). All 4 positive patients were male and 50–70 years of age (Supplemental Table 1).

**Fig. 1:**
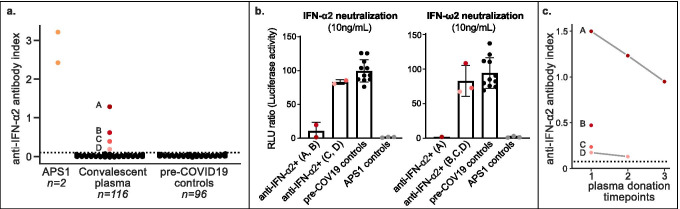
Anti-IFN-α2 antibodies in convalescent donor plasma. **A** anti-IFN-α2 radioligand binding assay identified 4 autoantibody positive convalescent plasma samples. Assay was repeated twice, with positive samples in duplicate. Dotted line indicates mean + 4 SD of pre-COVID-19 healthy controls. **B** Neutralization results from the 4 anti-IFN-α2 positive donors, 11 pre-COVID-19 healthy controls, and 3 APS1 positive controls. **C** Anti-IFN-α2 signal across multiple donation timepoints. Dotted line indicates mean + 4 SD of pre-COVID-19 healthy controls

We further tested these 4 samples for their neutralizing capacity against 10 ng/ml of IFN-α2 and IFN-ω in a cell-based assay. The sample with the highest signal (donor A) showed a complete neutralization capacity against both IFN-α2 and IFN-ω, while a second sample (donor B) showed neutralization against IFN-α2 only (Fig. 1b). Thus, 2/116 (1.5%) convalescent plasma samples from previously hospitalized donors had high titers of neutralizing auto-Abs against IFN-α2 and/or IFN-ω. Of the 4 unique donors, 2 had donated at multiple times and remained positive over all timepoints (Fig. 1c).

It remains to be determined whether administration of convalescent plasma containing type I interferon autoantibodies has any detrimental effect to patients, particularly when diluted in the recipients’ blood volume. However, in our original report, plasma from most patients could neutralize the protective effect of IFN-α2 against SARS-CoV-2 in vitro even when diluted up to 10,000-fold [4]. In light of recent reports on varying efficacy of convalescent plasma for COVID-19 treatment, it is worth considering that in rare circumstances, the presence of autoantibodies in the donor plasma pool could explain some of the variance in clinical response. Ongoing variation in the viral spike protein may necessitate the continued use of convalescent plasma, due to the slower pace of generation of recombinant monoclonal antibodies and possibly the emergence of vaccine-resistance [5]. While we previously found non-hospitalized donors to be anti-IFN-α2 negative [4], the data presented here suggest that COVID-19 convalescent plasma from previously hospitalized patients may require screening for type I interferon autoantibodies. Alternatively, COVID-19 plasma donation could exclude those donors with a record of severe to critical pneumonia until future studies can address whether transfer of autoantibodies constitutes harm to recipients.

## Materials and Methods

### Sample Selection and Acquisition

COVID convalescent plasma (CCP) samples were collected in the Vitalant system following FDA Guidance for donor eligibility as described in Vasallo et al. (2020). At the time of plasma collection, donors consented to use of de-identified donor information and test results for research purposes under the Vitalant Blood Donor Broad Consent based on the January 19, 2017, Final Common Rule, Federal Policy for the Protection of Human Subjects, judged exempt from IRB oversight by the Advarra IRB, Colombia, MD. The collection criteria evolved throughout the study period due to testing availability and evolution of the pandemic in the USA. Evidence of COVID-19 was required in the form of a documented positive SARS-CoV-2 molecular or serologic test, and complete resolution of symptoms initially at least 14 days prior to donation but then a minimum of 28 days was implemented. All CCP donors were also required to meet traditional allogeneic blood donor criteria. At the time of plasma collection, donors consented to use of de-identified donor information and test results for research purposes. All CCP were tested for SARS-CoV-2 total Ig antibody using the Ortho VITROS CoV2T assay at our central testing laboratory (Creative Testing Solutions [CTS], Scottsdale, AZ). CCP qualification requires the signal-to-cutoff ratio S/CO of this test to be at least 1.0. Retention samples of serum and plasma for all donations are archived at the Vitalant Research Institute Denver. Plasma from 116 unique donors identified as having a predonation history of hospitalization due to COVID-19 were tested. Vitalant collections were from June 5 to December 2, 2020.

APS1 positive control samples were previously published and collected as described in Ferre et al. (2016). All APS1 patients were enrolled in research study protocols approved by the NIAID, NIH Clinical Center, and NCI Institutional Review Board Committee and provided with written informed consent for study participation. All NIH patients gave consent for passive use of their medical record for research purposes (protocol #11-I-0187).

Healthy, pre-COVID control plasma were obtained from the New York Blood Center, where they were collected under informed consent, including usage for research.

### Anti-IFNA2 Radioligand Binding Assay

A sequence-verified plasmid containing full-length IFNA2 cDNA sequence with a Flag-Myc tag (Origene#RC221091) was used as template in T7-promoter-based in vitrotranscription/translation reactions (Promega, Madison, WI: #L1170) using [S35]-methionine (PerkinELmer, Waltham, MA; #NEG709A). Protein was column-purified using Nap-5 columns (GE Healthcare, Chicago, IL; #17–0853-01), incubated with 2.5-ul plasma or 1-ul anti-myc positive control antibody (CellSignal, Danvers, MA; #2272), and immunoprecipitated with Sephadex protein A/G beads (Sigma Aldrich, St. Louis, MO; #GE17-5280–02 and #GE17-0618–05, 4:1 ratio) in 96-well polyvinylidene difluoride filtration plates (Corning, Corning, NY; #EK-680860). The radioactive counts (cpm) of immunoprecipitated protein were quantified using a Microbeta Trilux liquid scintillation plate reader (Perkin Elmer). Antibody index for each sample was calculated as follows: (sample cpm value – mean blank cpm value)/(positive control antibody cpm value – mean blank cpm value). Positive signal was defined as greater than 4 standard deviations above the mean of pre-COVID-19 blood bank non-inflammatory controls.

### Neutralization Assays

HEK293T cells were transfected with firefly luciferase plasmids containing human ISRE promoters in the pGL4.45 backbone, and a constitutively expressing Renilla luciferase plasmid for normalization (pRL-SV40). Cells were transfected in the presence of the X-tremeGene 9 transfection reagent (Sigma-Aldrich) for 36 h. Medium was then removed and the cells were incubated for 16 hours with 10 ng/ml of IFN-a2 in presence of 10% of plasma from controls or from patients. Luciferase levels were measured with the Dual-Glo reagent, according to the manufacturer’s protocol (Promega). Firefly luciferase values were normalized against Renilla luciferase values, and fold induction is shown.

## Supplementary Information

Below is the link to the electronic supplementary material.

Supplementary file1 (PNG 56 KB)

## Data Availability

The data from this study are available upon request.
