# Supplementary figures and images for: Neutralizing Autoantibodies to Type I Interferons in COVID-19 Convalescent Donor Plasma
Source: J Clin Immunol. 2021 May 19;41(6):1169–71. doi: 10.1007/s10875-021-01060-0 (PMC8132742; doi:10.1007/s10875-021-01060-0)

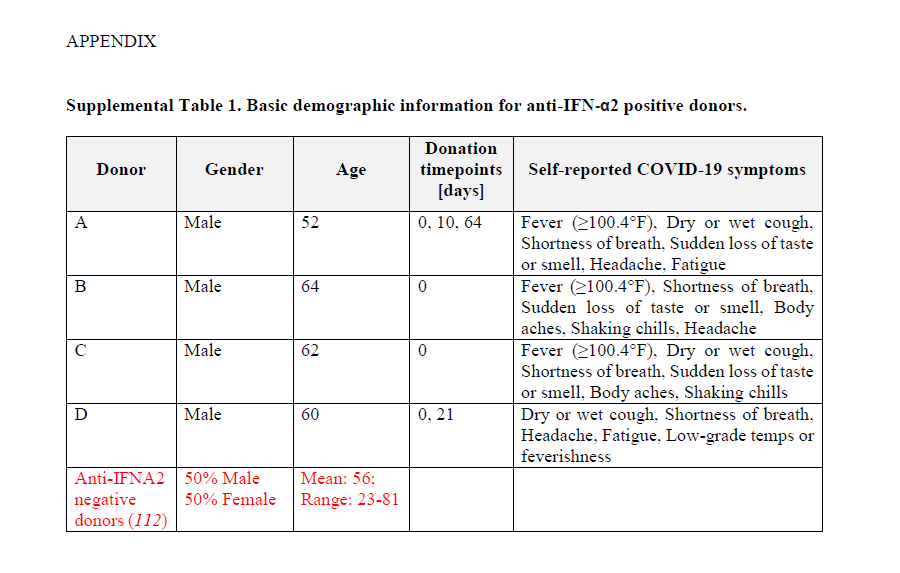

Supplement: Supplementary file 1 — Supplementary file1 (PNG 56 KB) [file 10875_2021_1060_MOESM1_ESM.png]
